# Removal of Pb^2+^, CrT, and Hg^2+^ Ions from Aqueous Solutions Using Amino-Functionalized Magnetic Nanoparticles

**DOI:** 10.3390/ijms232416186

**Published:** 2022-12-19

**Authors:** A. F. P. Allwin Mabes Raj, Maja Bauman, Marijana Lakić, Nena Dimitrušev, Aleksandra Lobnik, Aljoša Košak

**Affiliations:** 1 IOS, Institute of Environmental Protection and Sensors, Ltd., Beloruska 7, SI-2000 Maribor, Slovenia; allwinamc10@gmail.com (A.F.P.A.M.R.); maja.bauman@ios.si (M.B.); marijana.lakic@slu.se (M.L.); nena.dimitrusev@messergroup.com (N.D.); aleksandra.lobnik@um.si (A.L.); 2 Jožef Stefan International Postgraduate School, Jamova 39, 1000 Ljubljana, Slovenia; 3 Department of Environmental Science, Jožef Stefan Institute, Jamova 39, 1000 Ljubljana, Slovenia; 4 Faculty of Mechanical Engineering, Centre of Sensor Technology, University of Maribor, Smetanova 17, 2000 Maribor, Slovenia

**Keywords:** superparamagnetic nanoparticles, iron-oxide, maghemite, functionalization, aminopropyltrimethoxysilane, adsorption, desorption, lead, chromium, mercury, circular economy

## Abstract

In this paper, a circular economy approach with the adsorption and desorption of heavy metal (HM) ions—i.e., lead (Pb^2+^), chromium (CrT), and mercury (Hg^2+^)—from aqueous solutions was studied. Specific and selective binding of HM ions was performed on stabilized and amino-functionalized iron oxide magnetic nanoparticles (γ-Fe_2_O_3_@NH_2_ NPs) from an aqueous solution at pH 4 and 7. For this purpose, γ-Fe_2_O_3_@NH_2_ NPs were characterized by thermogravimetric analysis (TGA), Fourier-transform infrared spectroscopy (FTIR), specific surface area (BET), transmission electron microscopy (TEM), EDXS, and zeta potential measurements (ζ). The effects of different adsorbent amounts (m_ads_ = 20/45/90 mg) and the type of anions (NO_3_^−^, Cl^−^, SO_4_^2−^) on adsorption efficiency were also tested. The desorption was performed with 0.1 M HNO_3_. The results showed improvement of adsorption efficiency for CrT, Pb^2+^, and Hg^2+^ ions at pH 7 by 45 mg of g-Fe_2_O_3_@NH_2_ NPs, and the sequence was as follows: CrT > Hg^2+^ > Pb^2+^, with adsorption capacities of 90.4 mg/g, 85.6 mg/g, and 83.6 mg/g, respectively. The desorption results showed the possibility for the reuse of γ-Fe_2_O_3_@NH_2_ NPs with HNO_3_, as the desorption efficiency was 100% for Hg^2+^ ions, 96.7% for CrT, and 91.3% for Pb^2+^.

## 1. Introduction

Today, Europe is facing limited stocks of raw materials (RMs), such as heavy metal ions (HM ions) and rare-earth elements (REEs) [1,2,3,4], Even more obviously, in the context of the COVID-19 pandemic, Europe’s economy is facing an even larger lack of RMs and HM ions. Moreover, the European Union (EU)’s industry is dependent on imports of large amounts of RMs from the Asian market [1,2,3]. Therefore, the EU Commission was forced to prepare a list of critical raw materials (CRMs) [2,3,5,6], with sustainable strategies to foresee a circular economy based on recycling and reuse of critical REEs [2].

Lead (Pb^2+^), chromium (total chromium (CrT)), and mercury (Hg^2+^) ions are listed among the top 20 most hazardous substances [7,8] (accessed on 30 August 2022), since large amounts of HM ions are released into the environment due to agriculture and specific industries, such as the automotive, textile, mining, dye, and electroplating industries, among others [5,9,10,11]. HM ions dissolved in water are already toxic in small quantities and non-biodegradable, and some are carcinogenic and bioaccumulative, so they need to be treated as priority pollutants and efficiently cleaned [10,11,12]. 

Among these HMs, mercury has taken the spotlight because it is a global pollutant [13,14,15,16]. Mercury exists in various forms in the natural environment, such as mercurous (Hg_2_^+2^), and mercuric (Hg^2+^), along with organic mercury-containing methyl and ethyl groups. It is pertinent that the highly notorious form of methylmercury is caused by the methylation of inorganic [15] and elemental mercury [17] that is present in the aquatic environment by sulfate-reducing bacteria such as *Desulfovibrio desulfuricans*. Methylmercury can bioaccumulate and biomagnify in the oceanic food chain to reach 10^6^ times the concentrations that have caused several tragedies in the past, such as the Minamata tragedy in Japan [15] Negative toxic effects of versatile and highly mobile stable forms of chromium (Cr^3+^ and Cr^6+^) are a constant threat to humans and the environment. Depending on the pH, they can be present in acidic media (pH 0–4) in the form of soluble complexes (Cr^3+^, [Cr (H_2_O)_6_^3+^]), while near neutral (pH 6–9) inert precipitates (Cr (OH_3_) (s)) can easily be adsorbed on solid media [18].

HM ions are present in different concentrations (trace and shock concentrations) and forms [19,20], in combined industrial and municipal wastewater streams. Disposal of treated wastewater into the environment necessitates adjusting its pH to neutral. Especially for recycling of HM ions and water reuse [19,21], this means raising costs and increases the complexity of the pretreatment process, as well as increasing the addition of excessive amounts of chemicals [19,21].

Therefore, it is necessary to act sustainably and environmentally consciously by removing HMs from the highly polluted wastewaters using technology/methods that allow the removal/recycling of HM ions [4].

Currently, different conventional methods are used to remove HM ions from water/industrial wastewater [5,11], such as precipitation [22], electrochemical removal [23], ion exchange [24], membrane filtration [25,26], coagulation [27], flocculation [28], and sorption on natural materials [29]. Although these methods are efficient in removing HM ions, they do not allow the recycling and reuse of HM ions. Some already well-established methods for the removal of HM ions produce toxic coproducts and large amounts of waste sludge [30], e.g., membrane filtration and coagulation/flocculation [31]. Moreover, these methods are often costly and energy-inefficient [30].

On the other hand, the adsorption method is well known, efficient, and used for the removal of HM ions due to its low adsorbent and operational costs and simple principle [5,11,12]. Adsorption can be performed with various natural materials [29] and other hybrid materials based on silica and iron oxide NPs (γ-Fe_2_O_3_, Fe_3_O_4_), as well as their functionalized forms [32,33,34,35,36]. The most commonly used adsorbent of HM ions from industrial and leachate wastewaters is activated carbon [31]. Activated carbon is efficient in the removal of HM ions from wastewaters, due to its high specific surface area, micropore volume, and pore volume [31,33,37,38,39,40,41]. At the same time, limitations of its use include non-selectivity and high material price. Furthermore, activated carbon does not enable the recycling and regeneration of HM ions and the adsorbent itself, and for now it does not enable the circular economy approach [31,33]. 

Due to the increased need for recycling of municipal and industrial wastewaters [42] research in nanotechnology is investing in the preparation and testing of functionalized (nano)materials that can improve the recycling of specific HM ions [34,36,43,44]. 

Maghemite (γ-Fe_2_O_3_) is a member of the family of iron oxides. It has a cubic spinel ferrite structure, and it is ferrimagnetic. When reduced to particle dimensions smaller than a certain domain—i.e., becoming a single domain—ferrimagnetic materials exhibit superparamagnetic behavior, which means that when an external magnetic field is applied, they magnetize, but when the magnetic field is removed, they no longer exhibit either residual magnetism or coercivity. 

Such superparamagnetic nanoparticles, if they are surface-functionalized, provide promising applications in the adsorption of heavy metals from aqueous media, as they enable more efficient separation and recovery of heavy metals from the contaminated aqueous medium using an external magnetic field [45]. On the other hand, surface modification of superparamagnetic γ-Fe_2_O_3_ nanoparticles with TEOS and APTMS precursors improves their stability, prevents them from agglomerating, and increases their surface functionality by increasing the number of adsorption sites (-NH_2_), facilitating and accelerating diffusion pathways for heavy metal pollutants [46,47]. Despite all of the advantages of superparamagnetic γ-Fe_2_O_3_ nanocomposites for use in environmental technologies, the policy debate on their safety should not be ignored. Their toxicity is still an open question, even though much research has recently been carried out on this topic [48,49].

Surface functionalization of γFe_2_O_3_ nanoparticles was performed via a sol–gel method involving base-catalyzed hydrolysis and co-condensation of tetra-coordinated alkoxysilanes in an alcohol medium. Tetra-coordinated silanes can be described by the general chemical formula R’_x_Si(OR)_(4−x)_, 0 < x < 3, where OR is the hydrolyzable part (e.g., methoxy, ethoxy, etc.) and R’ is the non-hydrolyzable part of the structure with functional substituents (e.g., amino, mercapto, carboxy, etc.).

Ideally, it would be expected that the 3-aminopropyltrimethoxysilane (APTMS, (CH_3_O)_3_-Si-(CH_2_)_3_-NH_2_)) molecules on the surface of the γFe_2_O_3_ particles would polymerize into a highly homogeneous crosslinked SiO_2_ coating with functional amino (-NH_2_) groups present. However, the presence of a non-hydrolyzable fraction in the AMPTS structure ((CH_3_O)_3_-Si-(CH_2_)_3_-NH_2_)) causes steric hindrance, and the electron density on the silicon (Si) atom increases due to the inductive (+I) effect, which decreases the rate of hydrolysis and condensation of the APTMS and increases its tendency for homocondensation. The chemical reactivity is thus slowed down, leading to an undesired heterogeneous distribution of functional amino (-NH_2_) groups with an insufficient surface coverage of the γFe_2_O_3_ nanoparticles [50,51,52]. 

In contrast to APTMS, under base-catalyzed conditions, the reactivity of tetraethoxysilane (TEOS, Si(OCH_2_CH_3_)_4_) is enhanced due to the number and nature of the alkoxide (i.e., ethoxy) groups, which have a key influence on the crosslinking rate. This higher reactivity of TEOS can be attributed to the inductive stabilization of positively charged intermediates and transition states in the hydrolysis and condensation reactions by the ethoxy groups [53]. Therefore, TEOS was used as a crosslinker and APTMS ((CH_3_O)_3_-Si-(CH_2_)_3_-NH_2_)) was used as a supplier of the -NH_2_ functional groups. 

In this way, it was possible to create uniform spherical γFe_2_O_3_@SiO_2_-NH_2_ core–shell structures with the presence of amino (-NH_2_) functional groups on the surface of the nanoparticles, which are required for the subsequent binding of heavy metal ions from water [54].

The adsorption process of heavy metal ions for an adsorbent is highly dependent on the initial pH of the solution, owing to its remarkable effect on the speciation of metal ions [5].

If we take a closer look at the speciation of Cr, Pb, and Hg, we can find that at an acidic pH value, the predominant Cr(VI) species consist of H_2_CrO_4_^0^, HCrO_4_^−^, CrO_4_^2−^, and Cr_2_O_7_^2−^ [5,55] while Cr(III) remains relatively stable in acidic media and is more likely to be oxidized to chromate in alkaline media [56]. For Pb(II) in the pH range from 2 to 6, the dominant form is positively charged Pb^2+^ species, while when the pH values increase above 7, other Pb(II) species—including Pb(OH)^+^, Pb(OH)^2^, and PbO—are usually present [57]. 

Mercury has two common cations in aqueous solutions: a di-ion, Hg_2_^2+^, composed of two singly charged ions; and a doubly charged Hg^2+^. Diagrams of Eh-pH indicate that Hg(I) is stable only within a narrow band of Eh values in acidic solutions, while Hg(II) is the dominant form of the Hg species in most aqueous solutions [58]. The hydrolysis reactions of Hg(II) are significant at pH > 1, and different hydrolyzed forms can be formed depending on the aqueous mercury concentration [59] At low aqueous mercury concentrations, the dominant hydrolysis species formed are HgOH^+^ and Hg(OH)_2(aq)_, while at higher mercury concentrations the formation of Hg_2_(OH)_2_^2+^ and Hg(OH)^3–^ at pH > 13 has been reported [60].

Insoluble metal species will usually not form at pH < 7.2 as long as their concentration is below the solubility limit [61,62,63]. Therefore, at acidic pH values, the removal of positive heavy metal ions is mainly accomplished by adsorption. In contrast, at higher solution pH values, the precipitation of metal hydroxides or even oxides (e.g., Pb(OH)_2_, PbO, CrO_4_^2−^, etc.) can occur as a consequence of the low solubility of metal ions [57]. Therefore, at higher pH values, precipitation of insoluble species may take place at the same time alongside adsorption in the process of heavy metal removal, negatively affecting the adsorption efficiency [64]. 

Many studies have shown that metal ions start to precipitate as hydroxides or oxides when the solution pH is above 7.2. To avoid precipitation of the metal ions, all adsorption experiments should be conducted at a pH below 7.2 [56,61,62,63]. 

Moreover, the adsorption capacity of heavy metal ions decreases with increasing pH values. Specifically, it was shown that the maximum adsorption capacity of Cr(VI) is observed at a pH of 2 [5]. Moreover, the optimal pH for adsorbing Pb(II) was shown to be around 5.5 [65], whereas it was about 6 for Fe_3_O_4_@SiO_2_-NH_2_ magnetic nanoparticles [54,61]. 

Furthermore, it is generally known that iron oxides (γFe_2_O_3_, Fe_3_O_4_, etc.) suffer from a tendency to aggregate and decompose in acid-regenerated solutions; thus, to avoid the risk of potential dissolution of iron oxide cores at low pH, in this study, we instead used them in adsorption processes at pH > 3, despite silica shell protection (γFe_2_O_3_@SiO_2_-NH_2_) [61].

Iron oxide nanoparticles—i.e., goethite (α-FeOOH), hematite (α-Fe_2_O_3_), magnetite (Fe_3_O_4_), maghemite (γ-Fe_2_O_3_) [66,67,68,69,70], —show moderate affinity towards HM ions on their surface. They appear more applicable if the surface is stabilized [71,72,73], and enlarged by coating with silica NPs (-SiO_2_), whereby agglomeration is prevented [74]. Additionally, by using different functional groups [71,72,73]—e.g., amino (-NH_2_) [34,66,67,68,75,76], mercapto (-SH) [77,78], carboxy (-COOH) [79]—the adsorption efficiency and adsorption capacity of HM ions can be improved [33,34,71].

Adsorption studies of HM ions from model water by various magnetic nanoparticles (MNPs) and functionalized magnetic nanoparticles (F-MNPs) show that the maximum adsorption capacity of specific HM ions—i.e., for lead [44,61], mercury [80], and chromium [5,81]—can be obtained in less acidic pH.

In Table 1, Table 2 and Table 3, the adsorption capacity and desorption efficiency are compared for the tested MNPs and amino-functionalized MNPs at the optimal model solution pH values for adsorbing individual HM ions (e.g., Pb^2+^, CrT/Cr^3+^/Cr^6+^, and Hg^2+^).

Table 1 shows comparison of the adsorption capacities and desorption efficiency for Pb^2+^ ions by non-functionalized and functionalized MNPs. It can be seen that the adsorption of Pb^2+^ ions was tested mostly at acidic pH, and that the adsorption capacity is higher for the cases of functionalized magnetic nanomaterials. Ahmadi et al. (2014) [35] prepared γ-Fe_2_O_3_ NPs via the wet chemical method and tested adsorption at pH 7.5, while Nicola et al. (2020) [82] synthesized Fe_3_O_4_@SiO_2_ NPs and found that the adsorption capacity on non-functionalized MNPs was relatively low at pH 6.0 (10.55 mg/g) but a shade higher (14.9 mg/g) for SiO_2_-stabilized magnetic nanomaterials [82]. Nicola et al. (2020) [82] also tested the desorption efficiency of Pb^2+^ ions with 5% HCl, and the final desorption efficiency was evaluated as 95.7% [82]. Qian et al. (2019) [36] stated that the adsorption capacity of materials functionalized with chitosan and with an amino group (NH_2_-functionalized Fe_2_O_3_/chitosan NPs) at pH 5.0 was not significantly better compared to NH_2_-functionalized Fe_2_O_3_ materials. Higher adsorption capacity with Fe_3_O_4_ NPs coated with activated carbon was achieved at pH 6.0 [83], while Huang et al. (2020) [5] reached 53.9 mg/g with amino-functionalized graphene oxide at pH 5.0. Wang et al. (2010) [43] and Tang et al. (2013) [34] stated that even higher adsorption capacity at pH 6.2 (76.66 mg/g) can be achieved by pre-stabilization with SiO_2_ and amino-functionalization of magnetic materials (Fe_3_O_4_@SiO_2_–NH_2_ NPs). Tang et al. (2013) [34] achieved 82.29 mg/g with amino-functionalized Fe_3_O_4_@mesoporous SiO_2_ core–shell composite microspheres at pH 5.5. In polyethylenimine (PEI)-functionalized Fe_3_O_4_ magnetic nanoparticles (MNPs) (pH 5.0), adsorption capacity of 60.98 mg/g for Pb^2+^ ions was reported [84]. A maximum adsorption capacity of 60 mg/g at pH 5.0 was achieved using composite beads of *Zea mays* rachis (ZMR) and sodium alginate (AL) as adsorbents [85]. Luo et al. (2021) [86] reported the adsorption of 28.7 mg/g by carbon-doped TiO_2_ (C-TiO_2_) at pH 6.5 for the adsorption of Pb^2+^. The comparison of adsorption capacities showed that the adsorption capacity of Pb^2+^ ions depends on the pH of the medium, stabilization, and, to a large extent, the presence of -NH_2_ groups. 

From the literature, it can be observed that higher adsorption of Cr^3+^/Cr^6+^/CrT ions was achieved using amino (-NH_2_)-functionalized MNPs (Table 2). The highest adsorption of Cr^3+^ ions by bare MNPs was reported in the literature [72,87,88]. Zhang et al. (2020) [11] used bare magnetic magnetite NPs (Fe_3_O_4_) for the adsorption of Cr^3+^ ions at pH 4.0 and achieved an adsorption capacity of 8.67 mg/g. Additionally, in acidic media (pH 2.5), Gallo-Cordova et al. (2019) [72] performed adsorption of Cr^3+^ ions using bare iron oxide magnetic NPs and reported an adsorption capacity of 15.0 mg/g, while Hu et al. (2005) [17] achieved a very low adsorption capacity of 19.2 mg/g using maghemite NPs (γ-Fe_2_O_3_). Zhang et al. (2020) [11], Gallo-Cordova et al. (2019) [72], and Hu et al. (2005) [17] also performed desorption with NaOH, achieving desorption efficiency of >75%, ≅100%, and 87.7%, respectively. Other studies used amino-functionalized MNPs and achieved higher adsorption capacity in acidic media (pH 2.0 to 3.0). Adsorption of Cr^3+^ ions at pH 3.0 was performed using amino-functionalized magnetite NPs (NH_2_-Fe_3_O_4_) [89]. Baghani et al. (2016) [89] achieved an adsorption capacity of 24.25 mg/g and desorption efficiency of 98.02%. Even better adsorption (i.e., 35.0 mg/g) was reported by Gallo-Cordova et al. (2019) [72] using APTES@TEOS@MNP at pH 2.5, and the desorption efficiency was also high (≅100%). Zhao et al. (2010) [44] prepared NH_2_-functionalized nanomagnetic polymer adsorbents. Functionalization was performed with different precursors (i.e., EDA-, DETA-, TETA-, TEPA-). A maximum adsorption capacity of 38.5 mg/g at pH 2.5 was achieved using TETA-functionalized nanomagnetic polymer adsorbents. In another study, 40.0 mg/g of Cr^3+^ ions was adsorbed at pH 2.0 by TEPA-functionalized nanomagnetic polymer adsorbents [64]. Huang et al. (2020) [5] reported the adsorption properties of functionalized non-magnetic materials at pH 2.0. Using amino-functionalized graphene oxide (GO-NH_2_), Huang et al. (2020) [5] achieved 90.4 mg/g, which is the same adsorption capacity that we achieved with NH_2_-functionalized γ-Fe_2_O_3_ NPs (γ-Fe_2_O_3_@NH_2_ NPs), but at alkaline pH (7.0). The maximum adsorption capacity for chromium(VI) ions was 76.92 mg/g at pH 3.0 when adsorbent carbon-encapsulated hematite nanocubes (αFe_2_O_3_@C) were used [90]. Puszkarewicz and Kaleta (2019) [91] used activated carbon as an adsorbent, and the maximum adsorption capacity for chromium(VI) ions was 4.35 mg/g at pH 2 [91].

The maximum adsorption capacity for Hg^2+^ (Table 3) was 32.88 mg/g at pH 5.0 using carboxyl-terminated hyperbranched poly(amidoamine) dendrimers grafted onto superparamagnetic NPs (CT-HPMNPs) as adsorbents, and the maximum desorption efficiency was ≅85% (using HNO_3_ acid) [92]. Wang et al. (2013) [93] used rhodamine-hydrazide-modified Fe_3_O_4_ as an adsorbent, and the maximum adsorption capacity for Hg^2+^ was 37.4 mg/g at pH 7.5 [93]. Bolivar et al. (2018) [80] performed a study of Hg^2+^ ion adsorption, in which Fe_3_O_4_ nanoparticles coated with amino organic ligands and yam peel biomass displayed a maximum Hg^2+^ adsorption capacity of 60 mg/g at pH 7.0 [94]. The maximum adsorption capacity for Hg^2+^ was 50 mg/g at pH 7.0 when an adsorbent nanocomposite based on Fe_3_O_4_ nanoparticles, chitosan nanoparticles, and polythiophene was used [95]. Dun Chen et al. (2016) [96] studied the adsorption of Hg^2+^ using magnetic adsorbents (Fe_3_O_4_@SiO_2_-NH-HCGs; HCG = py (2-pyridinyl); pyd (3-pyridazinyl)) formed by grafting of different heterocyclic groups onto amino groups via substitution reaction. The maximum adsorption capacity for Fe_3_O_4_@SiO_2_-NH-HCG- (pyd) and Fe_3_O_4_@SiO_2_-NH-HCG- (py) was 77 mg/g and 56 mg/g at pH 7.0 [96], respectively. For both adsorption materials, HCl was used as the desorption eluent, and the stated desorption efficiency was 95% [96]. Hao et al. (2021) [97] performed a study of Hg^2+^ ion adsorption with Armeniaca sibirica shell activated carbon (ASSAC) magnetized by nanoparticles (Fe_3_O_4_/ASSAC), showing a maximum adsorption capacity of 97.1 mg/g at pH 2. At pH 5.5, Zhang et al. (2016) [98] studied the adsorption of Hg^2+^ ions with activated carbon (XLAC) derived from *Xanthoceras sorbifolia* Bunge hull as an adsorbent, showing a maximum adsorption capacity of 235.6 mg·g^−1^. A maximum adsorption capacity of 162 mg g^−1^ for Hg^2+^ ions at pH 5.0 was achieved using cadmium sulfide nanoparticles doped in a nanoadsorbent fabricated from polycaprolactam (nylon 6) nanofibers (CdS/N6) [99]. 

**Table 3:** Comparison of adsorption capacity and desorption efficiency for tested MNPs and amino-functionalized MNPs at optimal model solution pH for adsorbing Hg^2+^ ions.

Adsorbent	HM Ions	Tested pH	Adsorption Capacity	Desorption Efficiency	Reference
**NH_2_-functionalized γ-Fe_2_O_3_ NPs (γ-Fe_2_O_3_@NH_2_ NPs)**	Hg^2+^	**4.0**	**16.2 mg/g**	**100%**	**This work**
CT-HPMNPs		5.0	32.88 mg/g	≅85%	[92]
Rhodamine-hydrazide-modified Fe_3_O_4_		7.5	37.4 mg/g	-	[93]
Nanocomposite based on Fe_3_O_4_ nanoparticles, chitosan nanoparticles, and polythiophene		7.0	50 mg/g	-	[95]
Fe_3_O_4_@SiO_2_-NH-HCG- (py)		7.0	56 mg/g	95%	[96]
Fe_3_O_4_ nanoparticle coated with amino organic ligands and yam peel biomass		7.0	60 mg/g	-	[94]
Fe_3_O_4_@SiO_2_-NH-HCG- (pyd)		7.0	77 mg/g	95%	[96]
**NH_2_-functionalized γ-Fe_2_O_3_ NPs (γ-Fe_2_O_3_@NH_2_ NPs)**		**7.0**	**85.6 mg/g**	**100%**	**This work**
*Armeniaca sibirica* shell activated carbon (ASSAC) magnetized by nanoparticles (Fe_3_O_4_/ASSAC)		pH 2	97.1 mg/g		[97]
Activated carbon (XLAC) derived from *Xanthoceras sorbifolia* Bunge hull		pH 5.5	235.6 mg·g		[98]
Cadmium sulfide nanoparticles doped in a nanoadsorbent fabricated from polycaprolactam (nylon 6) nanofibers (CdS/N6)		pH 5	162 mg g		[99]

Adsorption has predominantly been investigated using -NH_2_ [36], -SiO_2_ [82], and -SH [94]-functionalized Fe_3_O_4_ or γ-Fe_2_O_3_ NPs [67] prepared by different approaches, in model water media of various pH values [34,66], from pH 2.0 to 8.0. 

There are not many previous studies [32,66] on testing adsorption by γ-Fe_2_O_3_ NPs functionalized with an amino (-NH_2_) group—specifically, by (3-aminopropyl)trimethoxysilane (APTMS) precursors—and to the best of our knowledge, far less research has been conducted on desorption approaches to date.

Although iron oxide and hybrid iron oxide NPs can be removed from aqueous solutions with an outer magnet, their recycling and regeneration possibilities after adsorption have not been sufficiently explored to fill gaps in the circular economy [32,47,87].

Due to these facts, our challenge was to synthesize and investigate the potential of amino-functionalized γ-Fe_2_O_3_ MNPs (γ-Fe_2_O_3_@NH_2_ NPs), which would allow efficient adsorption and recycling of HM ions at the shock load concentrations present in the model water, preferably at neutral pH, without pretreatment. To compare adsorption efficiencies and capacities, we tested significant concentrations of Pb^2+^, CrT, and Hg^2+^ ions using different amounts (m_ads_ = 20/45/90 mg) of the γ-Fe_2_O_3_@NH_2_ adsorbent NPs at two different pH values of the initial aqueous solution, i.e., at pH = 7.0, as well as at pH = 4.0. Furthermore, before the performance of adsorption tests, -NH_2_-functionalized γ-Fe_2_O_3_ MNPs were characterized with different methods, such as FTIR, BET, TEM, and TGA. Zeta potential changes in γ-Fe_2_O_3_@NH_2_ NPs were analyzed to understand the mechanisms taking place during the adsorption and desorption process of Pb^2+^ ions. Moreover, to evaluate the adsorbent regeneration, desorption with 0.1 M HNO_3_ was tested, which is of great importance for the reuse of adsorption materials and recycling of heavy metals. The prepared γ-Fe_2_O_3_ and functionalized γ-Fe_2_O_3_@NH_2_ MNPs were also characterized by X-ray powder diffractometry (XRD). 

## 2. Results and Discussion

### 2.1. Properties of the Prepared γ-Fe_2_O_3_@NH_2_ NPs

This section explains the characterization of the synthesized, stabilized, and functionalized MNPs (γ-Fe_2_O_3_@NH_2_ NPs). In addition, the adsorption mechanisms and the results of batch adsorption and desorption experiments are also discussed.

#### 2.1.1. Crystallographic Properties

**Figure 1:**
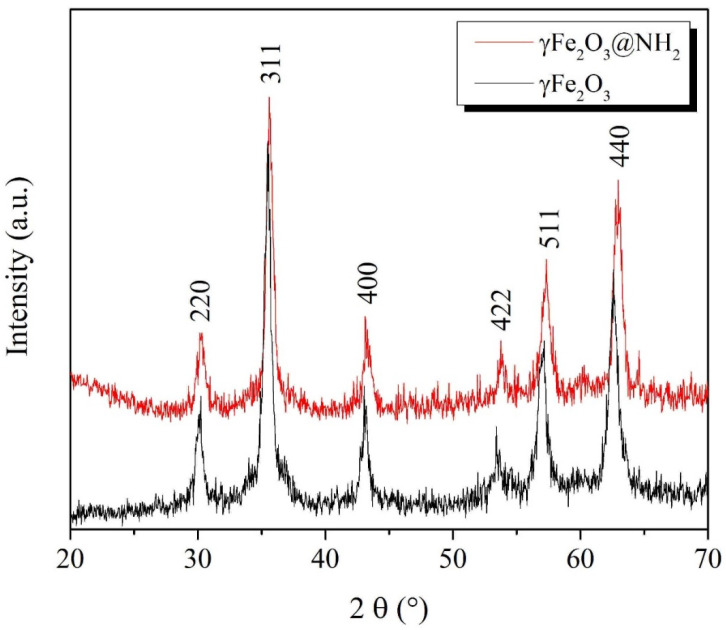
X-ray diffraction patterns (XRD) for the samples γ-Fe_2_O_3_ and γ-Fe_2_O_3_@NH_2_.

The prepared γ-Fe_2_O_3_ and functionalized γ-Fe_2_O_3_@NH_2_ MNPs were characterized by X-ray powder diffractometry (XRD) (Figure 1). In the X-ray powder diffraction pattern in Figure 1, the presence of diffraction peaks at 2*θ* of 30.2°, 35.5°, 43.2°, 53.6°, 57.2°, and 62.9°—which correspond to the cubic crystal planes of (220), (311), (400), (422), (511), and (440), respectively—are characteristic of the spinel crystal structure (JPCD Card 39-1346). The spinel crystal structure is evident for both samples—γ-Fe_2_O_3_ and functionalized γ-Fe_2_O_3_@NH_2_ MNPs—while the presence of a broad amorphous diffraction peak for the functionalized γ-Fe_2_O_3_@NH_2_ MNPs, which appears at a low diffraction angle 2*θ* of 20°, is due to the presence of the amorphous SiO_2_ surface layer, indicating that the crystalline cubic spinel γ-Fe_2_O_3_ magnetic cores were surface-modified [78]. The average size of the γ-Fe_2_O_3_ crystalline magnetic cores was estimated to be 13 nm, using the Debye–Scherrer equation [100,101].

#### 2.1.2. Thermogravimetric Properties

**Figure 2:**
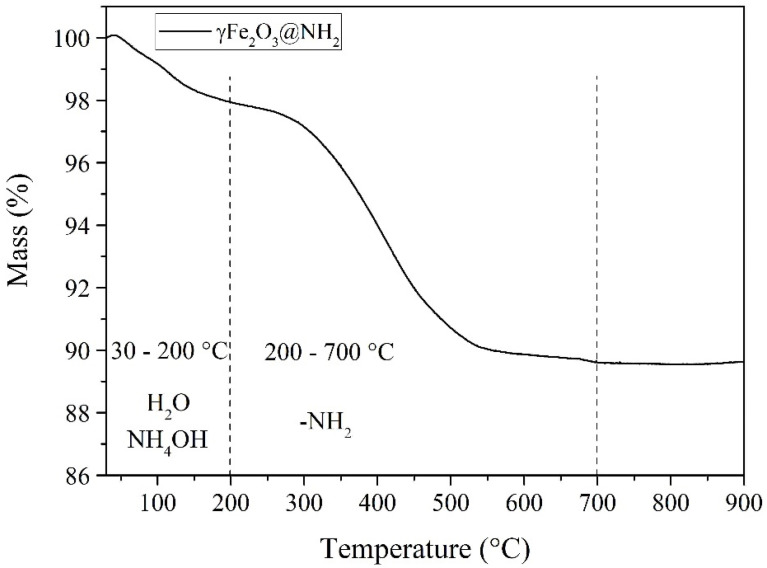
TGA analysis for γ-Fe_2_O_3_@NH_2_ NPs.

The thermal stability of γ-Fe_2_O_3_@NH_2_ NPs was determined via thermogravimetric analysis (TGA). The results of mass loss during the TGA analysis indicate the possible presence of -NH_2_ functional groups on the surface of the F-MNPs. Upon heating up to 180 °C, the measured mass loss corresponds to the evaporation of absorbed moisture and NH_4_OH residue. Further weight loss at heating up to 700 °C is due to the removal of aminopropyl (NH_2_(CH_2_)_3_-) groups from the nanoparticles’ surfaces and the consequence of cracking of the remaining siloxane groups (Si-O-Si) [75]. The TGA curve (Figure 2) shows that the synthesized, stabilized, and functionalized MNPs have good thermal stability. The weight loss during the TGA analysis was 10.3%.

The thermal stability of the particle samples analyzed was in accordance with previous results in the literature for other functionalized NPs [102,103,104,105]. 

#### 2.1.3. FTIR Spectroscopy

**Figure 3:**
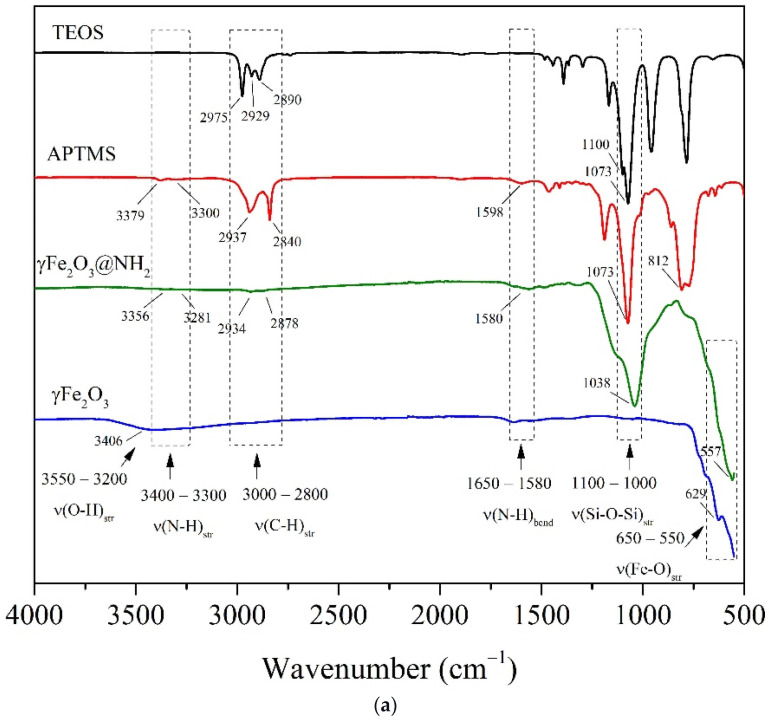

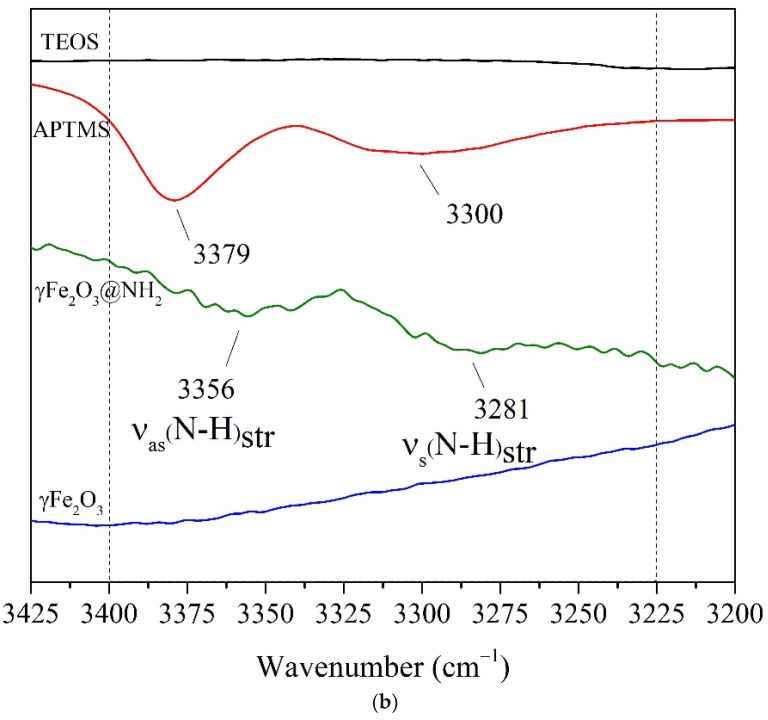
(**a**) FTIR spectra of γ-Fe_2_O_3_ NPs, γ-Fe_2_O_3_@NH_2_ NPs, pure AMPTS precursor, and pure TEOS precursor, and (**b**) enlarged area corresponding to vibrations of amino (-NH_2_) groups.

An FTIR analysis of γ-Fe_2_O_3_ and γ-Fe_2_O_3_@NH_2_ NPs was performed comparatively to identify the presence of characteristic functional groups related to the amino-silane coating of the γ-Fe_2_O_3_ surfaces. The FTIR spectra of γ-Fe_2_O_3_ and γ-Fe_2_O_3_@NH_2_ NPs, as well as those of pure TEOS and APTMS precursors, are shown in Figure 3a.

The functional amino-silane-coated γ-Fe_2_O_3_ nanoparticles were derived during the sol–gel process from the mixture of TEOS and APTMS precursors according to the experimental details described in Section 2.4. In contrast to the TEOS precursor (Si(OCH_2_CH_3_)_4_), the APTMS precursor ((CH_3_O)_3_Si(CH_2_)_3_NH_2_) included a short aliphatic chain (-(CH_2_)_3_-) and a terminal amino (-NH_2_) group in its structure. Thus, the main difference in the FTIR spectra of the TEOS and APTMS precursors is the presence of primary amino (N-H) vibrations in the range of 3400–3300 cm^−1^ of the APTMS spectra, while both spectra are identical to the occurrence of C-H vibrations in the range of 3000–2800 cm^−1^ and Si-O-Si vibrations in the range of 1100–1000 cm^−1^, which are common characteristics of alkoxysilanes.

As shown in Figure 3a, the formation of the γ-Fe_2_O_3_ structure is closely related to the occurrence of Fe-O bending and stretching vibrations in the range of 650–550 cm^−1^. The broad band at 3406 cm^−1^ observed for the γ-Fe_2_O_3_ NPs in the wavenumber region 3550–3200 cm^−1^ can be assigned to intermolecular O-H stretching (Figure 3a).

As opposed to γ-Fe_2_O_3_ NPs, asymmetric stretching vibrations of Si-O-Si bonds at 1050 cm^−1^ indicate the formation of a silica (SiO_2_) shell in the γ-Fe_2_O_3_@NH_2_ samples. Moreover, two weak bands can be observed for the γ-Fe_2_O_3_@NH_2_ samples in Figure 3a, characteristic of primary amines, due to the asymmetric and symmetric N-H vibrations in the range of 3400–3300 cm^−1^—more precisely, at 3356 cm^−1^ and 3281 cm^−1^, respectively. These primary amino peaks in the source spectra of γ-Fe_2_O_3_@NH_2_ NPs were not sufficiently visible, but enlarged individual peak areas confirmed their presence (Figure 3b). Specifically, the primary amine (NH_2_) vibrations occurred in the same wavenumber region as the intermolecular O-H stretching [106]. Because the polarity of the N-H bonds in amines is weaker than that of the O-H bonds, the absorption band of N-H is not as intense as that of O-H, which usually shows stronger and broader absorption bands that are much easier to identify. Primary amines have also a medium-to-strong absorption band in the wavenumber region 1650–1580 cm^−1^, which was identified at 1598 cm^−1^ for the γ-Fe_2_O_3_@NH_2_ NPs [107].

#### 2.1.4. Specific Surface Area

**Figure 4:**
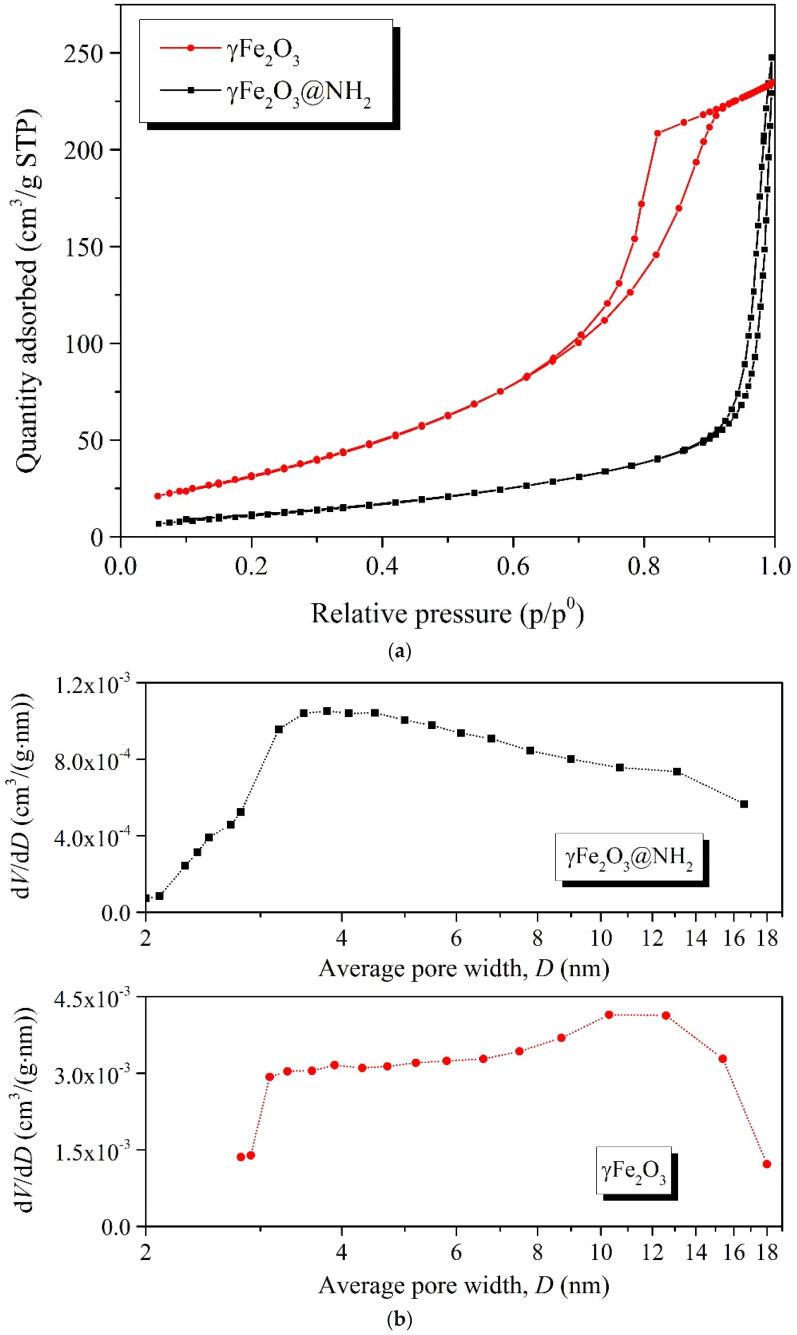
(**a**) BET isotherms and (**b**) pore size distribution for γ-Fe_2_O_3_ and γ-Fe_2_O_3_@NH_2_ NPs.

The specific surface area of the prepared γ-Fe_2_O_3_ and γ-Fe_2_O_3_@NH_2_ MNPs was measured by the Brunauer–Emmett–Teller (BET) method. The obtained BET curves are shown in Figure 4.

The BET analysis showed a specific surface area of 99.9 m^2^/g for γ-Fe_2_O_3_ and 41.3 m^2^/g for γ-Fe_2_O_3_@NH_2_. According to the Barrett–Joyner–Halenda (BJH) adsorption method, the average pore size was found to be 6.4 nm for the γ-Fe_2_O_3_ NPs, with a total pore volume of 0.378037 cm^3^/g, while for the BJH desorption the average pore size for the γ-Fe_2_O_3_ NPs increased to 6.7 nm, with a total pore volume of 0.407662 cm^3^/g, suggesting a mesoporous structure of the γ-Fe_2_O_3_ sample, with a typical type IV experimental N_2_ gas isotherm according to the IUPAC classification [108], as shown in Figure 4. In contrast to γ-Fe_2_O_3_, the γ-Fe_2_O_3_@NH_2_ sample showed a BET isotherm with a narrower hysteresis, indicating a decrease in the porosity of the as-prepared γ-Fe_2_O_3_ sample, most likely due to the presence of the homogeneous silicate coating. For BJH adsorption, the average pore size was found to be 5.8 nm for the γ-Fe_2_O_3_@NH_2_ NPs, with a total pore volume of 0.090762 cm^3^/g, while for the BJH desorption the average pore size for the γ-Fe_2_O_3_@NH_2_ NPs increased to 6.0 nm, with a total pore volume of 0.090311 cm^3^/g. 

According to the specific surface area (BET) at a relative pressure (p/p^0^) of 0.3, the calculated average particle size was 11.6 nm for γ-Fe_2_O_3_ and 27.9 nm for γ-Fe_2_O_3_@NH_2_ NPs [109,110] 

#### 2.1.5. Morphological Properties

**Figure 5:**
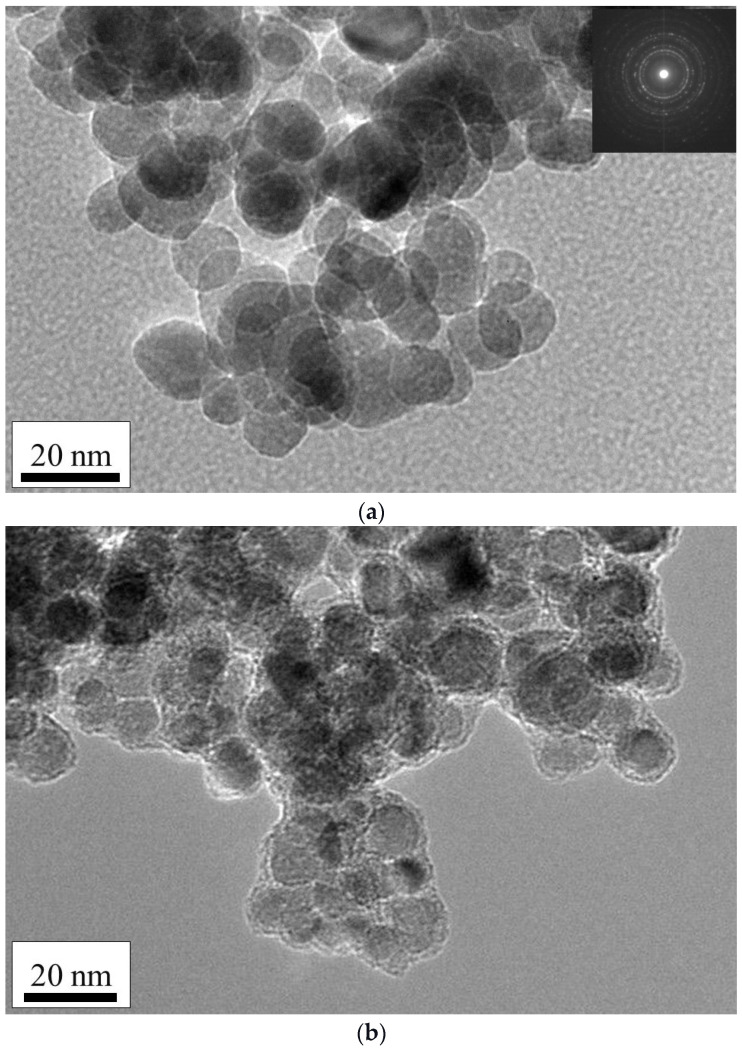
Transmission electron micrographs (TEM) of (**a**) γ-Fe_2_O_3_ NPs with inset diffraction pattern and (**b**) γ-Fe_2_O_3_@NH_2_ NPs.

The results of the TEM analysis (Figure 5a) represent the relatively spherical morphology of the γ-Fe_2_O_3_ MNPs, with a particle size distribution of 13 ± 1 nm, while the particle size distribution of the functionalized γ-Fe_2_O_3_@NH_2_ MNPs was 17 ± 1 nm (magnetic core 13 ± 1 nm and surface coating 4 ± 1 nm). The electron diffraction pattern of the γ-Fe_2_O_3_ MNPs inset in Figure 5b indicates the crystalline nature of the as-prepared powders, with concentric diffraction rings characteristic of a cubic spinel crystal structure. 

**Figure 6:**
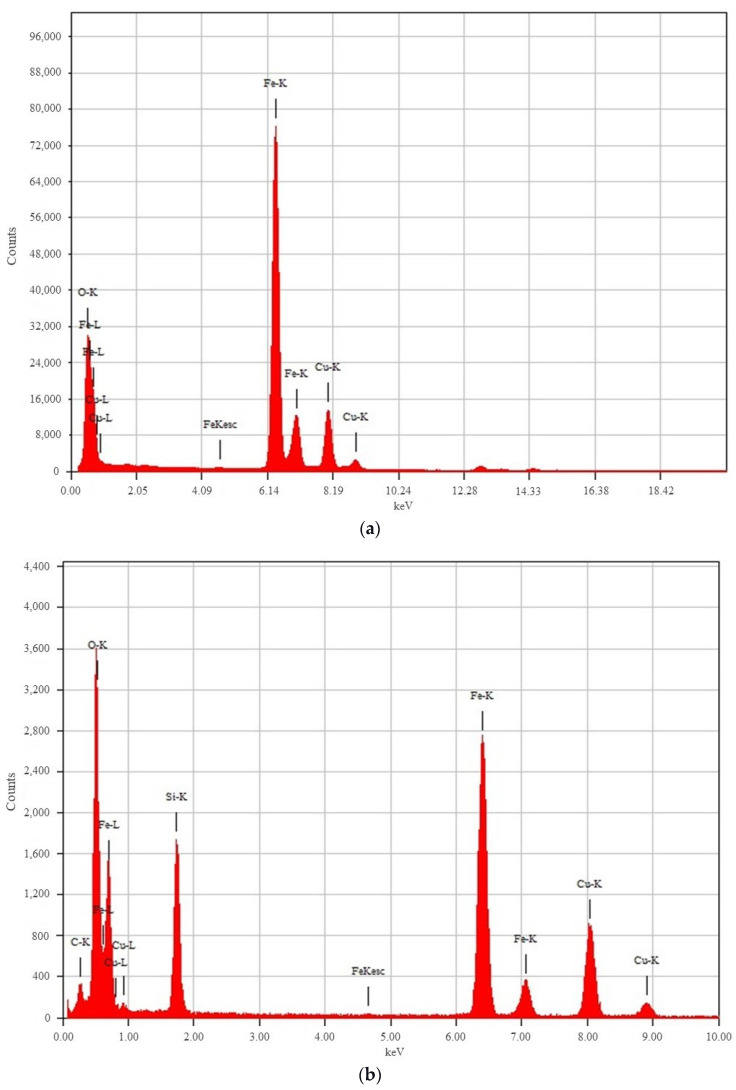
EDXS spectra of (**a**) γ-Fe_2_O_3_ NPs and (**b**) γ-Fe_2_O_3_@NH_2_ NPs.

The EDXS spectra of the γ-Fe_2_O_3_ and γ-Fe_2_O_3_@NH_2_ MNPs are shown in Figure 6a,b, respectively. Strong peaks for iron (Fe) and oxygen (O) can be seen in the EDXS spectrum in Figure 6a, indicating the formation of the γ-Fe_2_O_3_ MNPs. In contrast, the EDXS spectrum of the γ-Fe_2_O_3_@NH_2_ MNPs shows that they contain significant amounts of silicon (Si), alongside iron (Fe) and oxygen (O), suggesting the success of the surface functionalization of γ-Fe_2_O_3_ MNPs with APTMS precursor molecules and, thus, the formation of the γ-Fe_2_O_3_@NH_2_ MNPs. The lack of a nitrogen (N) peak is expected, due to its low Z-number and overlapping with the K-alpha C and O peaks. The larger peaks towards the right in both EDXS spectra are the copper (Cu) signals sourced from the TEM copper-grid-supported transparent carbon foil.

#### 2.1.6. Zeta Potential

**Figure 7:**
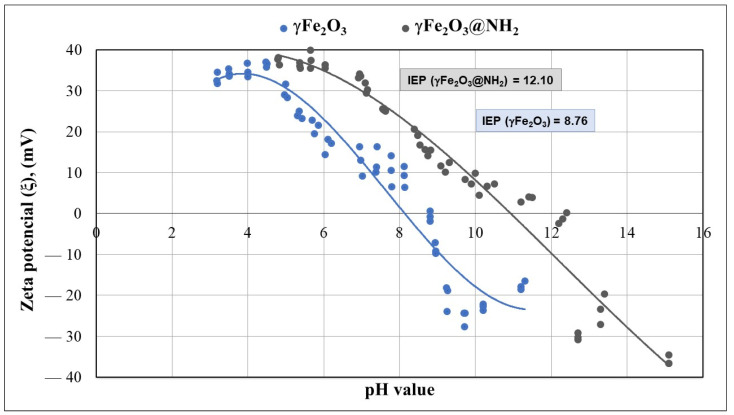
The zeta potential of bare γ-Fe_2_O_3_ and γ-Fe_2_O_3_@NH_2_ NPs.

The zeta potential was measured for bare MNPs (γ-Fe_2_O_3_) and amino-functionalized MNPs (γ-Fe_2_O_3_@NH_2_), as depicted in Figure 7. For bare, stabilized MNPs, the zeta potential is positive at low pH due to the presence of *OH*_2_^+^. As the pH of the solution increases, the potential decreases and approaches negative potential at high pH, due to the presence of *O*^−^. The measured isoelectric point of the bare MNPs was 8.76 (measured potential −0.743 mV). At this value, the concentration of protonated and deprotonated amino groups is the same. Meanwhile, the measured isoelectric point for functionalized MNPs was at pH 12.1 (measured potential +0.161 mV) =, indicating successful MNP functionalization. This difference in the isoelectric point is due to the presence of amino groups on MNPs, resulting in a functionalized magnetic nanomaterial with a negative charge above pH = 12.1.

### 2.2. Adsorption Mechanisms

The solution pH is a key parameter of the effectiveness of HM ions’ adsorption. HM ions have specific forms at different pH values; moreover, the adsorbent surface charge and protonation degree of the adsorbent surface coating (i.e., amino groups) are dependent on the pH [111,112]. In general, HM ions’ adsorption on γ-Fe_2_O_3_@NH_2_ NPs includes three sorption mechanisms, i.e., ion exchange, surface complexation, and electrostatic attraction [5]; the specific adsorption mechanism predominantly depends on the solution’s pH value [5].

We tested the adsorption of Pb^2+^, CrT, and Hg^2+^ ions at different pH values, i.e., pH 4 and 7. At different pH values, adsorption takes place by a different mechanism for each metal ion [5]. 

The adsorption of Pb^2+^ ions is entirely dependent on the pH value [70]. The adsorbent surface is negatively charged at alkaline pH, which indicates the deprotonated form of -NH_2_ functional groups. The behavior of -NH_2_ groups on the adsorbent material according to the pH is shown by Equations (1) and (2) [111]:

(1) $$-NH_{2}+H_{3}O^{+}\to -NH_{3}^{+}+H_{2}O$$

(2) $$-NH_{2}+OH^{-}\;\to -NH^{-}+H_{2}O$$

At pH 7, -NH_2_ groups are deprotonated, causing a negatively charged adsorbent surface, while lead ions are mostly in Pb (OH)^+^ form, which causes high electrostatic attraction between Pb^2+^ ions and the negatively charged material surface and, consequently, high adsorption efficiency [82,111]. On the other hand, acidic conditions cause the transformation of -NH_2_ groups into -NH_3_^+^ form, resulting in fewer available active sites for Pb^2+^ ions. Because of that, the adsorption efficiency of Pb^2+^ ions drops under acidic conditions (pH < 7) [5,111]. 

The solution pH value is also a key factor in the adsorption efficiency of CrT ions. Adsorption efficiency generally decreases with increasing pH values [5]. In acidic conditions, CrT ions are mainly present as H_2_CrO_4_^0^, HCrO_4_^−^, and Cr_2_O_7_^−^ species [68,72,81,113], while -NH_2_ is present in protonated form, i.e., -NH_3_^+^ form (Equation (3)). Consequently, the γ-Fe_2_O_3_@NH_2_ NPs’ surfaces are positively charged [5,81,114]. Strong electrostatic attraction occurs in such cases, and chromium species can be easily captured on the amino-functionalized adsorbent surface [72,81].

(3) $$R-NH_{2}+H^{+}\to R-NH_{3}^{+}$$

(4) $$-NH_{3}^{+}+HCrO_{4}^{-}\to -NH_{3}^{+}-HCrO_{4}^{-}$$

(5) $$-NH_{3}^{+}+Cr_{2}O_{7}^{2-}\to -NH_{3}^{+}-Cr_{2}O_{7}^{2-}$$

On the other hand, at alkaline pH, negatively charged chromate ions (CrO_4_^2−^) are the predominant form [81,87,113]. At pH > 7, γ-Fe_2_O_3_@NH_2_ NPs’ surfaces are also negatively charged [5] due to the deprotonated form of the amino functional groups. A double-negative charge of the adsorbent surface and chromate decreases the adsorption efficiency [72].

In our study, zeta potential played an important role in the adsorption mechanism. The zeta potential of our γ-Fe_2_O_3_@NH_2_ NPs was 8.76; at lower pH, amino groups on the material’s surface were mainly present in protonated form (-NH_3_^+^).

We tested the adsorption of CrT ions at pH 4 and 7. At pH 4, the functional groups were mainly in -NH_3_^+^ form, while at pH 7 the amino groups were still in protonated form. Consequently, many active sites were present on the surface of the γ-Fe_2_O_3_@NH_2_ NPs, so their adsorption capacity was very high. The adsorption efficiency at pH 4 was low due to the instability of γ-Fe_2_O_3_@NH_2_ NPs in acidic conditions—the adsorbent material is soluble in acidic media, i.e., at pH < 4.

Hg^2+^ readily reacts with OH^−^ to form Hg_2_(OH)_2_ precipitates under alkaline conditions. The adsorption of Hg^2+^ ions is predominantly influenced by the concentration of hydronium ions in aqueous solutions. The change in adsorption at varying pH levels is because the concentration of surface charges governs the adsorbent particles and the degree of ionization of the ions to be removed [111,115,116]. There is a variety of literature suggesting that the adsorption of Hg^2+^ ions favors neutral and basic pH. The rationale for more adsorption of Hg^2+^ ions using amino groups at neutral and basic pH is that the amino group obtains a net positive charge at acidic pH and the Hg^2+^ ions are also positive; hence, the adsorption is made unfavorable by the repulsive force. The above rationale for mercury species in aqueous solution was theoretically determined as a function of pH by modeling chemical equilibrium using MINEQL+ software (Environmental Research Software, Hallowell, ME, USA) [80,117].

### 2.3. Effects of pH

**Figure 8:**
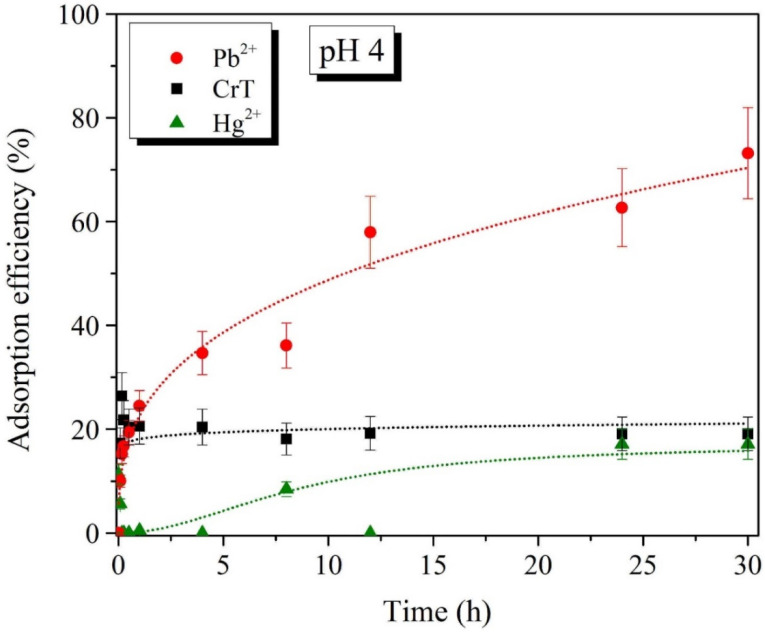
Adsorption efficiency (%) of Pb^2+^, CrT, and Hg^2+^ ions at pH = 4.

**Figure 9:**
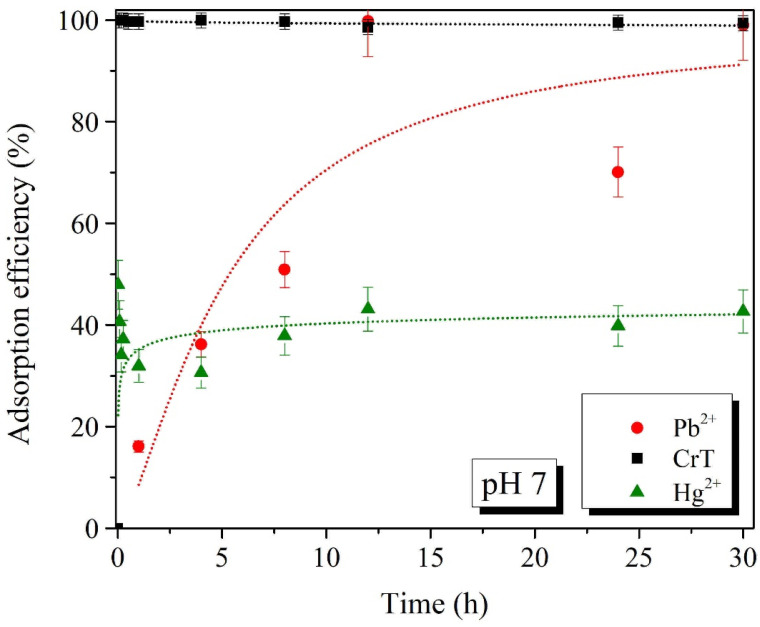
Adsorption efficiency (%) of Pb^2+^, CrT, and Hg^2+^ ions at pH = 7.

**Figure 10:**
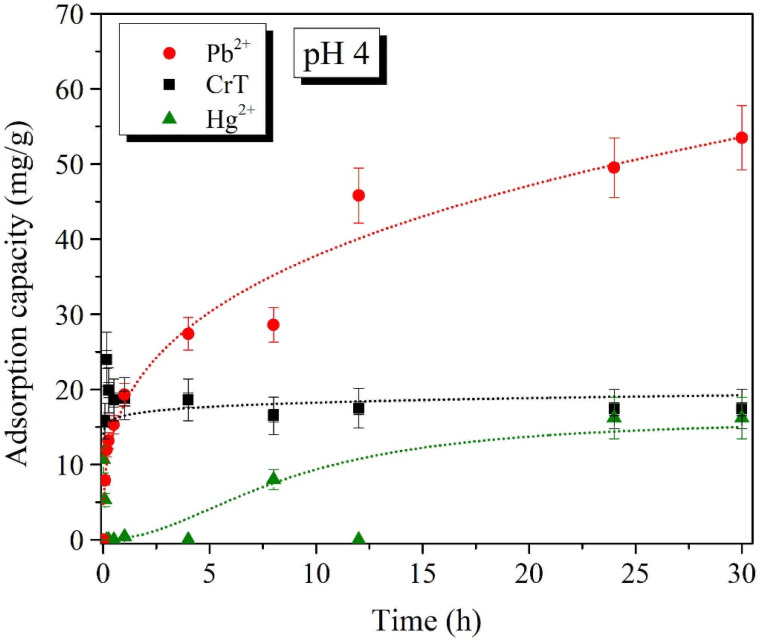
Adsorption capacity (mg/g) of Pb^2+^, CrT, and Hg^2+^ ions at pH = 4.

**Figure 11:**
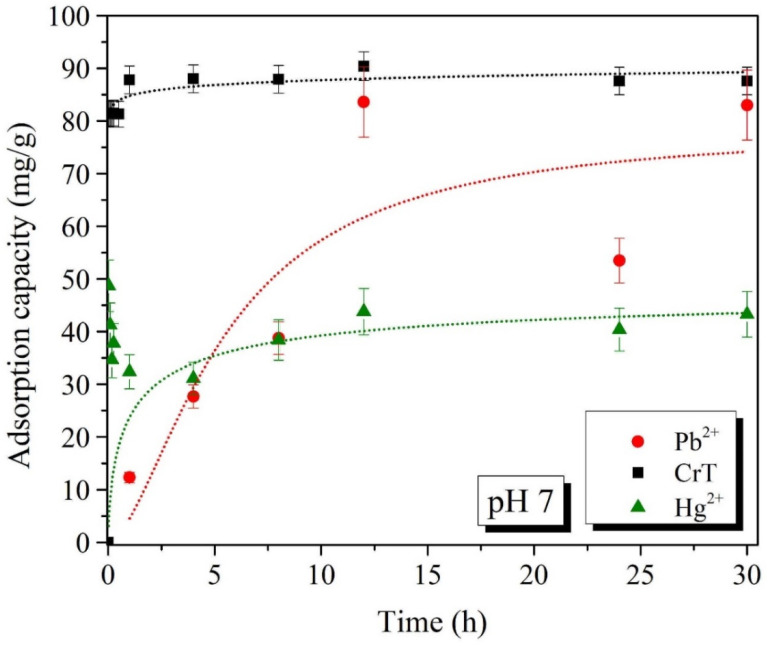
Adsorption capacity (mg/g) of Pb^2+^, CrT, and Hg^2+^ ions at pH = 7.

Batch adsorption experiments of Pb^2+^, CrT, and Hg^2+^ ions for different adsorption times were performed at two pH values, i.e., pH 4 and 7 (Figure 8, Figure 9, Figure 10 and Figure 11). The results show that the adsorption of Pb^2+^ and Hg^2+^ ions is more efficient at pH 7. Such results were expected, due to the opposite charges of the Pb^2+^ ions and the surface of the adsorption material. The opposite surface charges caused strong electrostatic interactions and high material uptake.

The adsorption capacity at pH 4 and 7 slowly increased with longer specific adsorption times. At pH 4, the maximal adsorption capacity of Pb^2+^ ions was 53.5 mg/g, which was detected after 30 h. At pH 7, the maximal adsorption capacity of 83.6 mg/g was achieved already after 12 h. At both tested pH values, the adsorption of Pb^2+^ ions slowly increased with a longer adsorption time. This indicates that the adsorption of Pb^2+^ ions is a slow process but, more importantly, the process is efficient—especially at pH 7.

Adsorption of CrT ions was much faster and very efficient at the same time. At pH 4, the adsorption efficiency was lower than 30%, and the maximal adsorption capacity was 24.0 mg/g. γ-Fe_2_O_3_@NH_2_ NPs were less stable in acidic conditions, which was the main reason for the lower material uptake. At pH 7, we achieved 99.9% adsorption efficiency already after 1 min. When the adsorption time was extended, the efficiency stayed high, and the maximal adsorption capacity (90.4 mg/g) was achieved after 12 h.

For Hg^2+^ ions, the maximal adsorption efficiency of 84.3% displayed a corresponding adsorption capacity of 85.6 mg/g, which was reached after 30 min of adsorption time. At pH 4, Hg^2+^ ions showed a low adsorption efficiency of 17%, with a corresponding adsorption capacity of 16.2 mg/g at 30 h. As demonstrated in various studies [80,111,115,116,117], the increase in pH from 4 to 7 also facilitated the maximal adsorption efficiency.

### 2.4. Effect of Adsorbent Mass

The adsorption of CrT ions on 45 mg of γ-Fe_2_O_3_@NH_2_ NPs showed excellent results (q_t_ after 1 min was 81.4 mg/g). To determine the optimal adsorbent mass, we tested different amounts of γ-Fe_2_O_3_@NH_2_ NPs. In adsorption experiments, 20, 45, and 90 mg of γ-Fe_2_O_3_@NH_2_ NPs were investigated under optimal adsorption conditions (pH = 7, c = 200 mg CrT/L and RT). Adsorption tests were performed only for 1, 4, 8, 24, and 30 h, as we expected that longer specific adsorption times would be required with smaller amounts of γ-Fe_2_O_3_@NH_2_ NPs.

**Figure 12:**
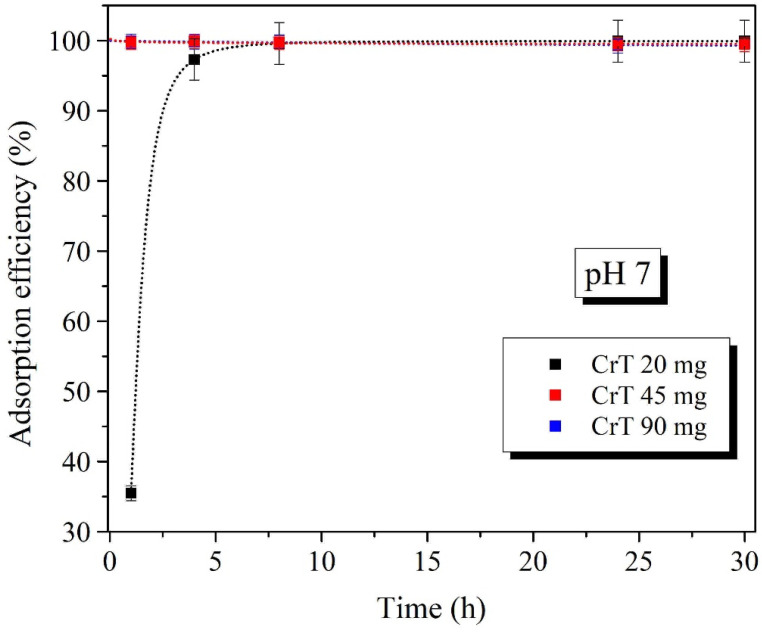
Adsorption efficiency of (%) CrT ions on m_ads_ = 20/45/90 mg pH = 7.

The results of CrT ions’ adsorption showed excellent adsorption efficiency (>99.2%) for all tested amounts of γ-Fe_2_O_3_@NH_2_ NPs at all tested specific adsorption times (Figure 12). The only exception was the test using 20 mg of γ-Fe_2_O_3_@NH_2_ NPs after 1 h. The adsorption efficiency on 20 mg of γ-Fe_2_O_3_@NH_2_ NPs was only 35.3%, indicating insufficient adsorbent mass. Nevertheless, the adsorption efficiency reached 99.9% after 24 h, showing that the adsorption of CrT ions with a smaller amount of γ-Fe_2_O_3_@NH_2_ NPs required a longer adsorption time. Meanwhile, the adsorption of CrT ions on 45 and 90 mg was equal; thus, based on the results of adsorption on 20/45/90 mg of γ-Fe_2_O_3_@NH_2_ NPs, we concluded that 45 mg was the optimal mass of adsorbent.

### 2.5. Effects of Anions (NO_3_^−^, Cl^−^, SO_4_^2−^)

The adsorption of CrT ions showed high adsorption efficiency (99.9%) after 1 min. For these reasons, we also tested the impacts of different anions (i.e., NO_3_^−^, Cl^−^, and SO_4_^2−^) on the adsorption efficiency of CrT ions. Experiments were performed under further optimal determined conditions (pH = 7, m_ads_ = 45 mg, c = 200 mg CrT/L), and only for 1, 5, 10, 15, 30, 45, and 60 min at RT.

**Figure 13:**
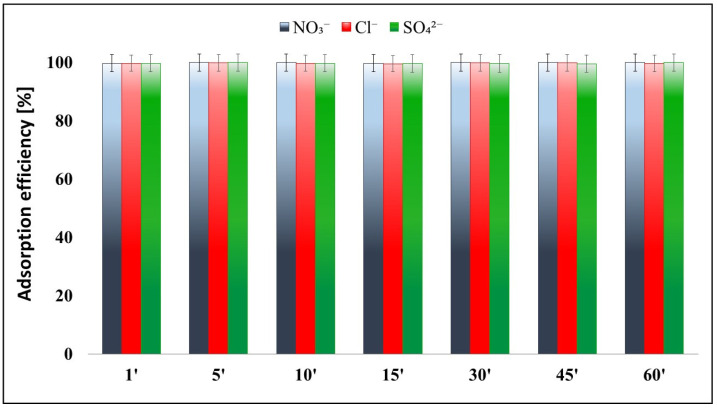
Effects of anions (NO_3_^−^, Cl^−^, and SO_4_^2−^) on the adsorption efficiency (%) of CrT ions pH = 7.

The results showed no effects of different anions; furthermore, the adsorption of CrT ions remained quick, and after 1 min the adsorption efficiency rate was 99.9% for all tested anions (Figure 13).

### 2.6. Desorption

**Figure 14:**
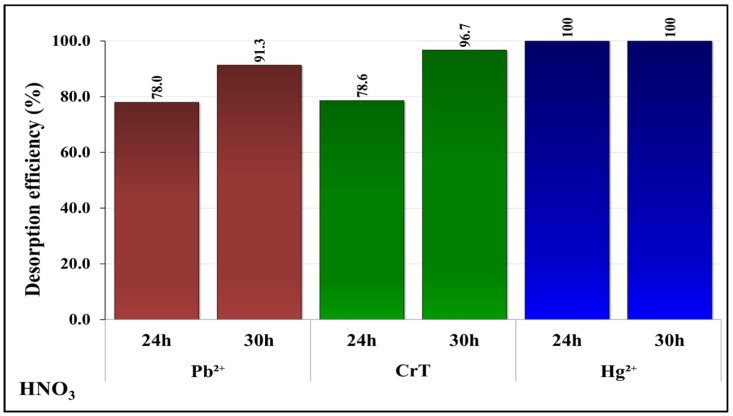
Desorption efficiency of Pb^2+^, CrT, and Hg^2+^ ions (after specific adsorption times at pH = 7). Desorption was performed with 0.1 M HNO_3_ for 60 min.

To verify the possibility of recycling HM ions and reusing adsorption materials, desorption of Pb^2+^, CrT, and Hg^2+^ ions was performed. Due to the higher adsorption capacity of metal ions at 1140 and 1800 min, adsorption was tested for longer specific adsorption times. Desorption was performed in one cycle because of material loss during the desorption process. The results of desorption showed that the γ-Fe_2_O_3_@NH_2_ NPs enabled high desorption efficiency for the samples on the surface of which the HM ions’ adsorption was performed at pH 7. This result indicates the electrostatic binding of HM ions on the adsorption material’s surface. Electrostatic binding of HM ions is weaker than covalent interactions, which probably appear at lower pH, i.e., pH 4. For this reason, only desorption results of samples after adsorption was performed at pH 7 are reported in this work (Figure 14).

The first desorption cycle performed with 0.1 M HNO_3_ was more efficient for Hg^2+^, CrT, and Pb^2+^ ions. For Pb^2+^ ions, the desorption efficiency was 91.3%; for CrT ions it was 96.7%; and for Hg^2+^ ions it was 100%. From the obtained results, it was possible to determine that higher desorption efficiency was achieved for all tested HM ions with a longer specific adsorption time (i.e., 30 h). 

The desorption efficiency of Hg^2+^ ions showed that the γ-Fe_2_O_3_@NH_2_ NPs enabled high desorption efficiency (100%) for all samples, with the adsorption process being carried out at pH 4 and 7.

## 3. Methods and Materials

### 3.1. Materials

For the lab-scale synthesis, stabilization, and functionalization of γ-Fe_2_O_3_ MNPs, iron(II) sulfate heptahydrate (FeSO_4_ 7H_2_O, 99.5%, 278.01 g/mol, CAS 7782-63-0, Honeywell International Inc., Charlotte, NC, USA), iron(III) sulfate hydrate (Fe_2_(SO_4_)_3_ xH_2_O, 97%, 399.88 g/mol, CAS 15244-10-7, Honeywell International Inc., Charlotte, NC, USA), tetraethyl orthosilicate TEOS (C_6_H_20_O_4_Si, 99%, 208.33 g/mol, CAS 78-10-4, Sigma-Aldrich, Merck Group KGaA, Darmstadt, Germany), (3-aminopropyl)trimethoxysilane APTMS (C_6_H_17_NO_3_Si, 97%, 179.29 g/mol, CAS 13822-56-5, Sigma-Aldrich, Merck Group KGaA, Darmstadt, Germany), ammonium hydroxide (NH_4_OH, 25%, 35.05 g/mol, CAS 1336-21-6, GramMol, Zagreb, Croatia), ethanol (C_2_H_5_OH, 96%, 46.07 g/mol, CAS 64-17-5, GramMol, Zagreb, Croatia), and 2-propanol (C_3_H_7_OH, 99.9%, 60.10 g/mol, CAS 67-63-0, GramMol, Zagreb, Croatia) were used. For the batch adsorption experiments, aqueous solutions were prepared from lead(II) nitrate (Pb(NO_3_)_2_, 99.9%, 331.21 g/mol, CAS 10099-74-8, Sigma-Aldrich, Merck Group KGaA, Darmstadt, Germany), chromium(III) nitrate nonahydrate (Cr(NO_3_)_3_ 9H_2_O, 99%, 400.15 g/mol, CAS 7789-02-8, Sigma-Aldrich, Merck Group KGaA, Darmstadt, Germany), mercury(II) nitrate monohydrate (Hg(NO_3_)_2_ H_2_O, ≥99.99%, 342.62 g/mol, CAS 7783-34-8, Sigma-Aldrich, Merck Group KGaA, Darmstadt, Germany), chromium(III) chloride hexahydrate (CrCl_3_ 6H_2_O, ≥98%, 266.45 g/mol, CAS 10060-12-5, Sigma-Aldrich, Merck Group KGaA, Darmstadt, Germany), and chromium(III) sulfate hydrate (Cr_2_(SO_4_)_3_ xH_2_O, 99%, 392.17 g/mol, CAS 15244-38-9, Sigma-Aldrich, Merck Group KGaA, Darmstadt, Germany). Chemicals were used as purchased. For the preparation of suspensions and solutions, deionized water (dH_2_O) was used.

### 3.2. Synthesis of MNPs

The synthesis of the maghemite (γ-Fe_2_O_3_) MNPs was carried out as described in our previous studies [32]. First, 30 mL of NH_4_OH was added to a 100 mL glass flask and heated up to 90 °C in an oil bath, under constant stirring at 220 rpm. Afterward, 50 mL of an aqueous solution prepared at a stoichiometric ratio of 1:2 using FeSO_4_ 7H_2_O and Fe_2_(SO_4_)_3_ xH_2_O salts was added to the reaction flask. Synthesis then proceeded in alkaline conditions at pH 10 for 1 h at 90 °C. After the reaction was finished, the suspension was cooled down to room temperature (RT), and the magnetic sediment was settled out for 30 min using an external permanent magnet. After magnetic separation, the supernatant was decanted and discarded. The colloid was rinsed several times with dH_2_O, centrifuged at 3500 rpm for 15 min and, finally, separated and allowed to settle on the external magnet overnight. 

### 3.3. Stabilization of MNPs

The rinsed γ-Fe_2_O_3_ MNPs were suspended in 100 mL of NH_4_OH overnight at RT under constant stirring (330 rpm) for the stabilization process. After 16 h, the stabilized maghemite MNPs were separated into two phases overnight on an external magnet. The upper phase was decanted, and the colloid was prepared for further functionalization.

### 3.4. Functionalization of MNPs

Amino-functionalization of the γ-Fe_2_O_3_ MNPs was carried out with tetraethyl orthosilicate (TEOS) and (3-aminopropyl)trimethoxysilane (APTMS) precursors, with a water-to-TEOS molar ratio R = 293 and TEOS-to-APTMS molar ratio P = 1:2. After the stabilization phase, 4 mL of γ-Fe_2_O_3_ colloidal solution was added to the mixture of 21.6 mol% 2-propanol, 2.2 mol% NH_4_OH, 15.1 mol% dH_2_O, 0.25 mol% TEOS, and 0.36 mol% APTMS, and then mixed vigorously for 5 min. After 24 h of reaction (at RT under constant stirring at 220 rpm), the colloidal solution was intensively washed with ethanol and dH_2_O and centrifuged at 3500 rpm for 5 min to remove agglomerates of non-functionalized γ-Fe_2_O_3_ MNPs.

### 3.5. Characterization of Amino-Functionalized γ-Fe_2_O_3_ MNPs

Characterization of the lab-scale amino-functionalized γ-Fe_2_O_3_ MNPs (γ-Fe_2_O_3_@NH_2_ NPs) was performed using the appropriate method after each preparation phase (i.e., synthesis, stabilization, functionalization). For characterization purposes, the γ-Fe_2_O_3_@NH_2_ MNPs were dried at 90 °C overnight, and the mass of the obtained dried particles was determined. The prepared γ-Fe_2_O_3_ and functionalized γ-Fe_2_O_3_@NH_2_ MNPs were characterized by X-ray powder diffractometry (XRD) (Bruker D4 Endeavor, Bruker, Billerica, MA, USA). The thermogravimetric properties were analyzed with a 4000 TGA PerkinElmer analyzer (PerkinElmer, Waltham, MA, USA), FTIR spectra were recorded with a Spectrum Two (PerkinElmer, Waltham, MA, USA), and specific surface area (BET) was measured with a TriStar II 3020 (Micromeritics Instrument Corporation, Norcross, GA, USA). The morphology of the synthesized γ-Fe_2_O_3_@NH_2_ MNPs was investigated using a transmission electron microscope (JEM 2100 JEOL, JEOL Ltd, Musashino Akishima, Tokyo, Japan) equipped with an energy-dispersive X-ray spectroscopy (EDXS) unit and a CCD camera to capture images.

### 3.6. Adsorption of Heavy Metal Ions in Aqueous Solutions

Batch adsorption tests of Pb^2+^, CrT, and Hg^2+^ ions were performed. The initial concentration of 200 mg/L of HM ions in the model water solutions was prepared by dissolving Pb (NO_3_)_2_, Cr (NO_3_)_3_ 9H_2_O, and Hg (NO_3_)_2_ H_2_O in a 1 L flask. The adsorption efficiency (R %) and adsorption capacity (q_t_ mg/g) at different pH of the model solution and adsorption at the defined time were calculated using Equations (6) and (7). Additionally, the effect of different adsorbent mass (m_ads_ = 20, 45, 90 mg) was tested, and the impact of various anions (e.g., NO_3_^−^, Cl^−^, and SO_4_^2−^) on the adsorption of CrT ions was investigated. For Pb^2+^ and Hg^2+^ ions, only adsorption at different pH values was tested.

The initial pH values of the solutions were measured and set to pH 4 with 0.1 M and 1 M HNO_3_ to simulate an acidic environment, which does not affect the γ-Fe_2_O_3_@NH_2_ NPs, while 1 M NaOH was used to adjust the pH to 7 to simulate actual wastewater conditions. Adsorption was conducted in 50 mL plastic centrifuges into which 20, 45, or 90 mg of the lab-scale γ-Fe_2_O_3_@NH_2_ NPs were weighed. Then, 20 mL of the prepared model salt solution was added to the γ-Fe_2_O_3_@NH_2_ NPs for selected specific adsorption times (1, 5, 10, 15, 30, 60, 240, 480, 720, 1140, and 1800 min). All tests were carried out at RT. To separate the γ-Fe_2_O_3_@NH_2_ NPs from the supernatant after adsorption, a centrifuge (4500 rpm, 15 min) (UNIVERSAL 320, Andreas Hettich GmbH & Co. KG, Tuttlingen, Germany) and an external magnet were used. The supernatant was decanted into a glass vial; meanwhile, the γ-Fe_2_O_3_@NH_2_ NPs were washed two times with 10 mL of dH_2_O.

The concentration of the HM ions in the supernatant was measured via atomic adsorption spectroscopy (AAS PerkinElmer, PerkinElmer, Waltham, MA, USA) and inductively coupled plasma optical emission spectrometry (ICP-OES, SPECTRO CITROS VISION, SPECTRO Analytical Instruments GmbH, Kleve, Germany) for Hg^2+^. For both analyses, the supernatants were acidified with HNO_3_ (0.5 mL of acid to 10 mL of the supernatant sample). 

The adsorption efficiency (R %) was calculated using Equation (6) [87]:

(6) $$R=\frac{\left(C_{0}-C_{t}\right)}{C_{0}}\times 100$$

where R (%) is the adsorption efficiency, C_0_ (mg/L) is the initial concentration of HM ions, and C_t_ (mg/L) is the residual concentration of HM ions. 

The adsorption capacity was calculated using Equation (7) [87]:

(7) $$q_{t}=\left(C_{0}-C_{t}\right)\times \frac{V}{m}$$

where q_t_ (mg/g) is the adsorption capacity, C_0_ (mg/L) is the initial concentration of HM ions, C_t_ (mg/L) is the residual concentration of HM ions, V (mL) is the solution volume, and m (mg) is the adsorption material mass.

### 3.7. Desorption of HM Ions and Regeneration Experiments

The desorption experiments for Pb^2+^, CrT, and Hg^2+^ ions were conducted to evaluate the recyclability of γ-Fe_2_O_3_@NH_2_ NPs. Desorption tests were performed immediately after specific adsorption times—i.e., 1, 1140, and 1800 min—and after rinsing twice with 10 mL of distilled water. Desorption was performed at RT with 20 mL of 0.1 M HNO_3_ added to 45 mg of adsorbent material. Desorption was in contrast to adsorption performed in dynamic mode with an IKA MS3 digital shaker (IKA-Werke GmbH & Co. KG, Staufen, Germany) at minimal rpm. The desorption efficiency was evaluated with AAS for Pb^2+^ and CrT, and with ICP-OES for Hg^2+^ ions.

## 4. Conclusions

In this study, stabilized and amino-functionalized magnetic nanoparticles (γ-Fe_2_O_3_@NH_2_ NPs) with a diameter of 17 ± 1 nm were synthesized, characterized, and used as adsorbents for Pb^2+^, CrT, and Hg^2+^ ions. Adsorbent characterization showed that γ-Fe_2_O_3_@NH_2_ NPs have good thermal stability. The particles were successfully stabilized, and amino-functionalization was confirmed with FTIR spectroscopy.

The adsorption process was carried out in aqueous solutions at pH 4 and 7. The adsorption results showed the highest adsorption efficiency and capacity at pH 7 for all investigated heavy metal (HM) ions, i.e., Pb^2+^, CrT, and Hg^2+^. The adsorption efficiency was the highest and quickest for CrT > Hg^2+^ > Pb^2+^ ions. The maximal adsorption capacity for Hg^2+^ ions was achieved in 30 min, at 85.6 mg/g; for CrT and Pb^2+^ ions, the maximal adsorption capacities were achieved after 12 h and were 90.4 mg/g and 83.6 mg/g, respectively. Experiments with different amounts of γ-Fe_2_O_3_@NH_2_ NPs (20/45/90 mg) showed that the optimal mass of adsorbent was 45 mg. Moreover, under optimal adsorption conditions (pH = 7, m_ads_ = 45 mg, c = 200 mg CrT/L, and RT), different anions—i.e., NO_3_^−^, Cl^−^, and SO_4_^2−^—showed no effect on the adsorption efficiency of CrT ions. 

A study of the desorption process with 0.1 M HNO_3_ for 1 h showed the possibility of reusing γ-Fe_2_O_3_@NH_2_ NPs. Desorption was effective only for γ-Fe_2_O_3_@NH_2_ NPs after the adsorption process was performed at neutral pH. We observed excellent desorption efficiency for Hg^2+^ (100%), CrT (96.7%), and Pb^2+^ (91.3%) ions.

The adsorption–desorption results showed that γ-Fe_2_O_3_@NH_2_ NPs have great ability and potential for specific and selective binding of HM ions and show excellent potential for real application in the circular economy. For this reason, further investigation of the circular adsorption–desorption process for different HM ions (such as copper, iron, and cadmium) in a single or binary system should be carried out in the near future.

The use of functionalized magnetic nanomaterials as adsorbents in this study showed that they combine the advantages of magnetic properties—which allow the removal of pollutants from water using an external magnetic field—with the properties of other functional materials, improving their adsorption, separation, and regeneration properties. Such adsorption materials are capable of removing the main components of inorganic pollutants, such as heavy metal ions, under different concentrations and pH conditions, due to their chemical and physical stability.

At the same time, this study showed that such functional magnetic nanoparticles, in conjunction with existing treatment technologies, can offer tremendous potential for the effective treatment of water and wastewater. Due to their unique properties related to magnetism and their surface and structural properties, these adsorption materials offer many alternative applications in many other fields. Their use has been growing in recent years, particularly in the recycling of critical materials—including rare-earth elements, which are now used in all high-tech products and are almost impossible to replace because their properties are unique or “rare”, which is why they are so highly valued, and their extraction and production pose major problems in terms of environmental pollution. In addition, such functionalized magnetic nanoparticles could also be effectively used to remove organic and biological pollutants such as organic dyes, fluoridated and chlorinated organic compounds, pesticides, bioactive compounds, etc., which are often found in groundwater, drinking water, and wastewater.

Despite the vast potential shown by functionalized magnetic nanomaterials as adsorbents, most of them are still at the laboratory research stage. The lack of legislation and regulation and the issue of toxicity of nanomaterials, which should also not be ignored, represent the major obstacles encountered in the remediation of water and wastewater with nanomaterials, while many other obstacles associated with their use are only temporary, such as high costs and technical handling. 

Although many studies have been carried out on the adsorption of heavy metal ions, the mechanism of their interaction with adsorbents is, in many cases, not fully understood. Therefore, more research on the interactions between functionalized magnetic nanomaterials and pollutants is expected in the future, as they are of key importance for the design and improvement of the properties of adsorbents, but the lack of knowledge on their environmental and human impacts has to be taken into account in order to move towards a justification of their use in real environments.

## Figures and Tables

**Table 1:** Comparison of adsorption capacity and desorption efficiency for tested MNPs and amino-functionalized MNPs at the optimal model solution pH for adsorbing Pb^2+^ ions.

Adsorbent	HM Ions	Tested pH	Adsorption Capacity	Desorption Efficiency	Reference
γ-Fe_2_O_3_ NPs	Pb^2+^	7.5	10.55 mg/g	-	[35]
Fe_3_O_4_@SiO_2_ NPs		6.0	14.9 mg/g	95.7%	[82]
NH_2_-functionalized Fe_2_O_3_/chitosan NPs		5.0	32.46 mg/g	-	[36]
NH_2_-functionalized Fe_2_O_3_ NPs		5.0	39.30 mg/g	-	[36]
Magnetic composite of activated carbon and superparamagnetic Fe_3_O_4_ NPs (Fe_3_O_4_@C magnetic composite)		6.0	41.7 mg/g	>77%	[83]
**NH_2_-functionalized γ-Fe_2_O_3_ NPs (γ-Fe_2_O_3_@NH_2_ NPs)**		**4.0**	**53.5 mg/g**	**90.7%**	**This work**
Amino-functionalized graphene oxide (GO-NH_2_)		5.0	53.9 mg/g	-	[4]
Fe_3_O_4_@SiO_2_–NH_2_ NPs		6.2	0.37 mmol/g 76.66 mg/g *	-	[43]
Amino-functionalized Fe_3_O_4_@mesoporous SiO_2_ core-shell composite microspheres		5.5	82.29 mg/g	-	[35]
**NH_2_-functionalized γ-Fe_2_O_3_ NPs (γ-Fe_2_O_3_@NH_2_ NPs)**		**7.0**	**83.6 mg/g**	**91.3%**	**This work**
Polyethylenimine (PEI)-functionalized Fe3O4 magnetic nanoparticles (MNPs)		pH 5.0	60.98 mg/g		[84]
Composite beads of *Zea mays* rachis (ZMR) and sodium alginate (AL)		pH 5.0	60 mg/g		[85]
Carbon-doped TiO_2_ (C-TiO_2_)		pH 6.5	28.7 mg/g		[86]

* calculated.

**Table 2:** Comparison of adsorption capacity and desorption efficiency for tested MNPs and amino-functionalized MNPs at optimal model solution pH for adsorbing CrT/Cr^3+^/Cr^6+^/Cr(VI) ions.

Adsorbent	HM Ions	Tested pH	Adsorption Capacity	Desorption Efficiency	Reference
Magnetic magnetite NPs (Fe_3_O_4_)	CrT/Cr^3+^/Cr^6+^/Cr(VI)	4.0	8.67 mg/g	>75%	[87]
Iron oxide magnetic NPs (MNPs)		2.5	15.0 mg/g	≅100%	[72]
Maghemite NPs (γ-Fe_2_O_3_)		2.5	19.2 mg/g	87.7%	[88]
**NH_2_-functionalized γ-Fe_2_O_3_ NPs (γ-Fe_2_O_3_@NH_2_ NPs)**		**4.0**	**24.0 mg/g**	**-**	**This work**
Amino-functionalized magnetite NPs (NH_2_-Fe_3_O_4_)		3.0	24.25 mg/g	98.02%	[89]
APTES@TEOS@MNP		2.5	35.0 mg/g	≅100%	[72]
NH_2_-functionalized nanomagnetic polymer adsorbents (EDA-NMPs)		2.5	37.6 mg/g	-	[44]
NH_2_-functionalized nanomagnetic polymer adsorbents (DETA-NMPs)		2.5	37.9 mg/g	-	[44]
NH_2_-functionalized nanomagnetic polymer adsorbents (TETA-NMPs)		2.5	38.5 mg/g	-	[44]
NH_2_-functionalized nanomagnetic polymer adsorbents (TEPA-NMPs)		2.0	40.0 mg/g	-	[44]
Amino-functionalized graphene oxide (GO-NH_2_)		2.0	90.4 mg/g	-	[4]
**NH_2_-functionalized γ-Fe_2_O_3_ NPs (γ-Fe_2_O_3_@NH_2_ NPs)**		**7.0**	**90.4 mg/g**	**96.7%**	**This work**
Carbon-encapsulated hematite nanocubes (αFe2O3@C)		pH 3	76.92 mg/g		[90]
Activated carbons		pH 2	4.35 mg/g		[91]
